# A Tale of Two Joints: The Role of Matrix Metalloproteases in Cartilage Biology

**DOI:** 10.1155/2016/4895050

**Published:** 2016-07-13

**Authors:** Brandon J. Rose, David L. Kooyman

**Affiliations:** Department of Physiology and Developmental Biology, Brigham Young University (BYU), LSB 4005, Provo, UT 84602, USA

## Abstract

Matrix metalloproteinases are a class of enzymes involved in the degradation of extracellular matrix molecules. While these molecules are exceptionally effective mediators of physiological tissue remodeling, as occurs in wound healing and during embryonic development, pathological upregulation has been implicated in many disease processes. As effectors and indicators of pathological states, matrix metalloproteinases are excellent candidates in the diagnosis and assessment of these diseases. The purpose of this review is to discuss matrix metalloproteinases as they pertain to cartilage health, both under physiological circumstances and in the instances of osteoarthritis and rheumatoid arthritis, and to discuss their utility as biomarkers in instances of the latter.

## 1. Introduction

Matrix metalloproteinases are a family of zinc-dependent endopeptidases collectively capable of degrading all components of the extracellular matrix. The actions of these enzymes are potent and highly catabolic, and as such physiologic expressions of the genes coding for matrix metalloproteinases are strictly regulated and reserved for instances where dramatic tissue remodeling is required, as occurs during wound healing [1] and embryonic development [2]. Their versatility and efficacy also render them potent effectors of pathological processes, and this is where much interest in their activity is garnered. Ectopic overexpression matrix metalloproteinase activity has been implicated in a wide array of disease states, including tumor initiation and metastasis, atherosclerosis, osteoarthritis, and rheumatoid arthritis. The purpose of the present review is to discuss matrix metalloproteinases as they relate to articular cartilage homeostasis.

## 2. The Role of Matrix Metalloproteinases in Healthy Cartilage

Seven matrix metalloproteinases have been shown to be expressed under varying circumstances in articular cartilage—matrix metalloproteinase-1 (MMP-1), matrix metalloproteinase-2 (MMP-2), matrix metalloproteinase-3 (MMP-3), matrix metalloproteinase-8 (MMP-8), matrix metalloproteinase-9 (MMP-9), matrix metalloproteinase-13 (MMP-13), and matrix metalloproteinase-14 (MMP-14). Of those seven, four have been found to be constitutively expressed in adult cartilage, presumably serving roles in tissue turnover, and upregulated in diseased states—MMP-1, MMP-2, MMP-13, and MMP-14 [3]. The presence of the MMP-3, MMP-8, and MMP-9 in cartilage appears to be characteristic of pathologic circumstances only.

MMP-1 (interstitial collagenase) is involved in the degradation of collagen types I, II, and III. In embryonic development its expression is restricted to areas of endochondral and intramembranous bone formation and is especially abundant in the metaphyses and diaphysis of long bones. During that time, it is expressed in hypertrophic chondrocytes (immediately preceding terminal differentiation in endochondral ossification) and osteoblasts only [4]. Expression levels are low under healthy circumstances, but significant upregulation is observed in arthritic cartilage and may play an active role in collagen degradation in this tissue but is evidently absent in the instance of synovitis [5].

MMP-2 (gelatinase A) is involved in the breakdown of type IV collagen and is most commonly expressed early in the process of wound healing [6]. Expression in adult cartilage is weak and attributable to normal (very low) collagen turnover, and, similar to MMP-1, it is upregulated in arthritic states [7].

MMP-3 (stromelysin-1) is capable of degrading a wide array of extracellular molecules, including collagen types II, III, IV, IX, and X, fibronectin, laminin, elastin, and various proteoglycans. In addition, it has been found to have transcription factor-like activity, apparently being able to upregulate the expression of other matrix metalloproteinases [8]. It is involved in wound healing, expression being typical in fibroblasts and epithelial cells following expression to inflammatory compounds [9], possibly explaining the presence of high MMP-3 levels in osteoarthritic cartilage and the synovium in osteoarthritis [10] and absence in normal joint tissues and showing promise for this enzyme as a candidate marker for osteoarthritis [11].

MMP-8 (neutrophil collagenase) is the principal collagenase found in human dentin, being involved in turnover and remodeling in that tissue [12], and it is expressed in a wide array of cell types, including neutrophil precursors and epithelial cells [13]. Consistent with most other matrix metalloproteinases, it is involved principally in wound healing, mostly in wounds of an acute character [14]. Its expression in arthritic tissue is clearly beneficial; genetic deficiencies of MMP-8 exacerbate inflammation in arthritis through downregulation of neutrophil apoptosis and clearance, subsequently causing hyperinfiltration of joints with neutrophils [15].

MMP-9 (gelatinase B), similar to MMP-1, is most active during embryonic development, being essential to angiogenesis in the growth plate and apoptosis of hypertrophic chondrocytes in utero [16]. It has also been demonstrated to be highly expressed in the early stages of wound healing [17], perhaps due to its involvement in angiogenesis. In the joint capsule it is produced by monocytes and macrophages; production by chondrocytes appears minimal [18], though these cells do appear to play a considerable regulatory role in leukocyte MMP-9 expression. Inhibition of leukocyte release by chondrocytes appears to be lifted in an arthritic state, apparently in response to increased MMP-3 activity (possibly via transcriptional regulation) and MMP-13 expression [19].

MMP-13 is by far the most studied of the matrix metalloproteinases in terms of its role in cartilage, as it is considered the major catabolic effector in osteoarthritis and other forms of arthritis, owing to its robust ability to cleave the type II collagen that predominates in articular cartilage. Having such a unique and dramatic effect on said tissue, it is obvious why MMP-13 is frequently employed as the matrix metalloproteinase of choice in the detection and study of osteoarthritis. Particularly telling and supporting its utility as a biomarker in osteoarthritis is the tendency of external influences to act upon the joint through upregulation of MMP-13 specifically, as will be discussed in further detail later in this review. Though as is mentioned it is involved principally in the degradation of type II collagen, the enzyme also targets other matrix molecules such as types IV and IX collagen, perlecan, osteonectin, and proteoglycan [20], and it is likely involved in matrix turnover in healthy cartilage.

MMP-14 (membrane-type 1 matrix metalloproteinase) is involved in aggrecan degradation and cadherin cleavage and has been shown to be involved in inhibition of tumor angiogenesis [21]. It has been shown to have a significant role in postnatal bone formation through promotion of osteogenesis and chondrogenesis [22]. MMP-14 is upregulated in arthritic cartilage and furthermore appears to have the ability to activate MMP-2 [23] and MMP-13 [24], potentially compounding its influence in arthritis.

The careful modulation of matrix metalloproteinases is required for maintenance of cartilage health. The presence of these enzymes alone does not constitute pathology; as indicated, deficiencies in MMP-8 are deleterious to joint health; rather a careful balance is required for maintenance of the anabolic/catabolic balance, and it is dysregulation that brings about the catabolism of articular cartilage in arthritic disease.

Having discussed the role of matrix metalloproteinases in healthy cartilage, it is now pertinent to discuss their overexpression and the relation of this phenomenon to disease states, beginning with osteoarthritis and following with rheumatoid arthritis.

## 3. The HTRA1-DDR2-MMP-13 Axis

As indicated, several matrix metalloproteinases are upregulated and known to play a role, beneficial or deleterious, in osteoarthritis and as such are candidate biomarkers in osteoarthritis. Nevertheless, we look to one in particular, MMP-13, as the principal effector of cartilage degradation in osteoarthritis and the most obvious and useful candidate for matrix metalloproteinases as a biomarker in the disease.

Common to the pathogenesis of osteoarthritis in known processes culminating in the condition is the activation of the HTRA1-DDR2-MMP-13 axis. High temperature requirement A1 (HTRA1), a serine protease, is strongly expressed in the presence of stressors in murine osteoarthritis models. HTRA1 is responsible for degradation of pericellular matrix components, including fibronectin, matrilin 3, collagen oligometric matrix protein, biglycan, fibromodulin, and type VI collagen [25]. Breakdown of the pericellular matrix exposes the chondrocyte membrane to the type II collagen characteristic of articular cartilage, activating and augmenting the expression of the transmembrane discoidin-containing domain receptor 2 (DDR2) [25]. Heightened expression of DDR2 results in excess binding of the receptor to its ligand [26] in turn stimulating high levels of expression of the MMP-13 gene, culminating in the extracellular matrix and leading to the destruction of articular cartilage [27, 28]. Aside from the wide array of evidence showing HTRA1-DDR2-MMP-13 activity in osteoarthritis of multiple modalities, the convergence of diverse noxious stimuli upon this axis is further supported in the protective effects exerted in DDR2 hypomorphic strains of mice, which, when subject to the DMM procedure, demonstrated a significant decrease in the progression of osteoarthritis compared to wild type littermates [29], comparable to the ablation of MMP-13, which has the effect of protecting cartilage from degradation in osteoarthritis induced through destabilization of the medial meniscal ligament (DMM-induced osteoarthritis) in murine models, though interestingly enough chances to the chondrocytes themselves and other cells in response to the procedure persisted [17].

## 4. Molecular Pathways Associated with Transcriptional Regulation of MMP-13 Gene Expression

Gene expression of MMP-13 appears to occur through a number of molecular pathways that work through either inflammation or primary cilia. That is not to say there are not some common themes. Stress-inducible nuclear protein 1 (Nupr1) has been shown to regulate MMP-13 expression in vitro [30]. Yammani and Loeser showed that Nupr1, expressed in cartilage, is required for expression of MMP-13 via IL-1*β*. This might be a pathway for the catabolic effects of OA to be mediated through inflammation. This is especially interesting in light of the study done by Xu et al., 2015, in which they analyzed differential expression of genes in cartilage involved in OA and RA [31]. While these researchers identified multiple genes associated with the regulation of MMPs, the predominant ones were associated with inflammation. This might give greater credence for the role of early inflammatory signals (i.e., AGEs and IL-1) in the initiation and progression of OA. Meanwhile more obviously, a similar role for inflammation appears to be present in RA. Araki et al., 2016, reported that histone methylation and the binding of signal transducer activator of transcription 3 (STAT3) were associated with RA and OA [32]. They report that histone H3 methylation is associated with elevated expression of MMP-1, MMP-3, MMP-9, and MMP-13. However, STAT3 was shown to increase expression, either spontaneous or IL-6 activated, of MMP-1, MMP-3, and MMP-13 but not MMP-9. As previously indicated, primary cilia appear to also be involved in OA. Sugita et al., 2015, reported that transcription factor hairy and enhancer of split-1 (Hes1) is involved in the upregulation of expression of MMP-13 [33]. Normally Hes1 acts as a transcriptional repressor but under the influence of calcium/calmodulin-dependent protein kinase 2 (CaMK2) it becomes a transcriptional activator, thus upregulating MMP-13 expression [34]. Thus Hes1 acts to increase expression of MMP-13. It is of particular interest to note that Hes1 acts through Notch signaling pathway [35]. Notch has previously been shown to modulate sonic hedgehog signaling and work through primary cilia [36, 37]. In an apparent unrelated mechanism, Niebler et al., 2015, showed that the transcription factor AP-2*ϵ* is intimately involved in the upregulation of MMP-13 as OA progresses [38].

## 5. Metabolic Syndrome and Upregulation of Matrix Metalloproteinases

An area of study in the field of rheumatology that presents ample opportunity for research and great promise for therapeutic intervention involves the interaction between articular cartilage and metabolic (insulin resistance) syndrome. The comorbidity between osteoarthritis and metabolic syndrome and its individual manifestations is striking—epidemiological data reveals that 49% of individuals with heart disease, 47% with diabetes, 44% with hypertension, and 31% of obese individuals also have some form of arthritis [39], suggestive of a common or overlapping etiology between osteoarthritis and these conditions. Further incriminating a load-bearing hypothesis of osteoarthritis has been the determination that hand osteoarthritis is more strongly correlated with body mass index than hip osteoarthritis, the latter of which was found to have such a weak relationship as to not being statistically significant [40].

The opportunities for interaction between metabolic syndrome and osteoarthritis are vast and cross a temporal spectrum spanning from an initial hyperinsulinemic state [41] to proper insulin resistance [42] to the downstream effects of metabolic syndrome, including but not limited to type II diabetes mellitus [43], a decrease in circulating HDL particles [44], and high circulating levels of adipokines.

While hyperinsulinemia is implicated in the chondrocyte apoptosis facet of osteoarthritis [41], expression of MMP-13 has not been documented to occur until the point of insulin resistance in the joint capsule. In one study, mice fed a high fat diet to generate the obese/type II diabetes mellitus (ob/t2d) phenotype showed considerably increased levels of tumor necrosis factors (TNFs) in the synovial fluid of the knee joint, and studies comparing ob/t2d with TNF knockout mice demonstrated that TNF species were linked to increased expression of MMP-1, MMP-13, and ADAMTS4—a mouse homologue of matrix metalloproteinases. Supplementation with insulin in these mice inhibited these effects by 50% [42].

Leptin, a peptide hormone involved in maintaining insulin sensitivity and contributing to the sensation of satiety, is expressed at very high levels in obese individuals. It appears to be correlated with osteoarthritis as well, with intervention at the level of MMP-13 expression occurring. Downregulation of leptin mRNA translation via small interference RNA molecules inhibits MMP-13 expression in cultured osteoarthritic chondrocytes [45]. The situation appears to be most exacerbated in cases of extreme obesity; there exists a strong positive correlation between the responsiveness of the MMP-13 gene to leptin and the BMI of osteoarthritic individuals [46]. Its effects are not limited to MMP-13 alone; MMP-1 expression and MMP-3 expression are strongly upregulated in osteoarthritic cartilage, and leptin levels strongly correlated with the presence of these two matrix metalloproteinases in osteoarthritic synovial fluid [47]. Adiponectin also appears to have some ability to stimulate expression of MMP-13. The presence of adiponectin is positively correlated with the presence of membrane-associated prostaglandin E2 synthase (mPGES) and MMP-13 [48]. Furthermore, cultured osteoarthritic chondrocytes treated with adiponectin showed increases in production of nitric oxide via inducible nitric oxide synthase (iNOS), expression of MMP-1, MMP-3, and MMP-13, and levels of collagenase-cleaved type II collagen neoepitope. These effects were attenuated in the presence of AMP-activated protein kinase (AMPK), c-Jun N-terminal kinase, and iNOS inhibitors, implicating these as mediators of adiponectin-induced insult [49].

Hyperglycemia is linked to the presence of high levels of circulating advanced glycation end products, particularly of the S100 family of proteins [50]. This phenomenon is linked to an array of diabetes-induced complications, including atherosclerosis, and a deficiency in the receptor for advanced glycation end products (RAGE) proves to have protective effects, implicating this receptor in the pathway. Ablation of RAGE in osteoarthritic murine models likewise shows a protective effect; wild-type mice, on the other hand, demonstrate increased expression of proinflammatory markers and catabolic mediators, including MMP-13 [51]. RAGE is known to act through a host of proinflammatory means, including NF-*κ*B, TNF, IL-1, and TGF-*β* [52], and though it has not been conclusively demonstrated, there is abundant potential for a catabolic role of RAGE in metabolic osteoarthritis.

## 6. Matrix Metalloproteinases in Response to Mechanical Insult

Activation of matrix metalloproteinases, especially MMP-13, is known to occur in response to mechanical injury to the joint, and factors leading to its expression are far more clear-cut than in metabolic osteoarthritis. In response to injury to the joint, the first response that appears to be elicited from chondrocytes and synoviocytes secretes interleukin-1*β*. This in turn activates the cell surface receptor IL-1RI, which initiates a cascade of intracellular events involving NF-*κβ*, MAPK, p38, and JNK. This results in increased expression of TNF-*α*, which further potentiates inflammatory events already in play [53] and initiates chondrocyte expression of MMP-1 [54], MMP-2 [55] and MMP-13 [56].

Over the course of this process, chondrocytes begin to express the cell proliferant transforming growth factor-*β* (TGF-*β*) [57], perhaps in response to its ability to mitigate some of the activities of IL-1*β* and produce additional chondrocytes to cope with stressors placed on the joint. Overexpression of this factor, however, appears to somehow be involved in initiating the osteoarthritic process [58]. This elevation in TGF-*β* corresponds to an increase in HTRA-1 [59], perhaps in consequence of the ability of the latter to cleave the former [60], and consequentially DDR2 is activated and MMP-13 is highly expressed. The body of evidence implicates MMP-13 as a major effector of mechanically mediated joint destruction in osteoarthritis.

Consistent with previously discussed studies, DDR2 is shown to be a major effector of MMP-13 expression in DMM models, such that almost complete protection from osteoarthritis is shown in DDR2 hypomorphic strains. It is also telling to note that genetic ablation of the receptor for advanced glycation end products (RAGE) corresponds to a decreased expression of MMP-13 in DMM models, though not as dramatic as DDR2 hypomorphism, and that this decreased expression corresponds with decreased progression and severity of osteoarthritis. This suggests a contributing role of RAGE in the pathogenesis of osteoarthritis and certainly a target for therapeutic intervention. Conversely and for reasons that are not yet completely understood, pharmacological antagonism of the proinflammatory transmembrane protein Toll-like receptor 4 (TLR-4) results in heightened MMP-13 expression and a corresponding dramatic increase in the severity of osteoarthritis [61]. Further research is required to elucidate a rationale for this phenomenon, and this information must be considered in devising therapeutic intervention for acute joint injury with the goal of delaying or preventing osteoarthritis development later in life.

## 7. Genetic Anomalies Resulting in Matrix Metalloproteinases Overexpression and Osteoarthritis

As stated, a number of disease states involve overexpression of matrix metalloproteases, and there exist diseases in which mutations result in overexpression of MMP-13 and premature development of osteoarthritis. One such abnormality is spondyloepiphyseal dysplasia congenita (SEDC), which serves as an experimental model of osteoarthritis. In SEDC, a mutation occurs in the COL2A1 gene, resulting in the formation of superfluous disulfide bridges in type II collagen, adversely affecting association between individual collagen molecules and thus the triple helix that is characteristic of the typical collagen molecule [62]. It is possible that this failure of collagen monomers to form proper interactions predisposes the individual to premature osteoarthritis rendering the molecules ideal targets for the binding and activity of HTRA1 and DDR2 and downstream to these MMP-13 explaining the dramatically increased levels of each characteristic of the syndrome [4].

Another disease that has been the focus of osteoarthritis research as of late is the ciliopathy Bardet-Biedl syndrome (BBS). BBS may result from mutations in a number of the known genes that are involved in ciliary formation and transport [63], resulting in a number of congenital abnormalities including polydactyly, cognitive impairment, cardiac and renal malformation, obesity hypertension, and type II diabetes mellitus. In addition, mouse models of BBS manifest early onset osteoarthritis; whether this is a direct result of ciliary malformation or secondary to osteoarthritis risk factors such as obesity and type II diabetes mellitus is presently unclear. What is known is that, in BBS osteoarthritic cartilage, TGF-*β* is downregulated, HTRA1 is upregulated, and MMP-13 is strongly expressed in the absence of DDR2. This finding strongly suggests a role of primary cilia in DDR2 activity and/or signal transduction, and further research is clearly warranted to elucidate the link between cilia and DDR2.

## 8. Matrix Metalloproteinases and Rheumatoid Arthritis

Rheumatoid arthritis is a form of arthritis in which the host immune system mounts an attack on connective tissue in the joint. MMPs are associated with rheumatoid arthritis [64]. In their study, Ahrens et al. demonstrated that MMP-9 is elevated in the synovial fluid of patients with rheumatoid arthritis. Indeed, they indicated that SF levels of MMP-9 were higher in rheumatoid arthritis patients compared to osteoarthritis. It is of interest to note that they also speculated that elevated MMP-9 was associated with connective tissue turnover in rheumatoid arthritis patients.

These observations led to the intriguing possibility of determining the severity of rheumatoid arthritis by examination of matrix metalloproteinases in blood. Keyszer et al. were the first to show a correlation between blood circulating matrix metalloproteinases and the clinical manifestation of rheumatoid arthritis [65]. They demonstrated that circulating levels of MMP-3 were a better predictor of rheumatoid arthritis severity or activity than cytokine level. These observations led to the thinking that perhaps clinicians could separate the immunological and inflammatory mechanisms associated with RA from the actual erosion of articular surface. Uncoupling these two pathophysiological pathways may aid in new treatment regimens and strategies. Indeed, Cunnane et al. were the first to make this connection [66]. They showed that the degree of articular surface erosion correlated with levels of MMP-1 and MMP-3. They concluded that treatments for rheumatoid arthritis which specifically target MMP-1 may limit the number of new joint erosion foci and thus improve the overall functional outcome for RA patients.

Gender can be a complicating issue for both osteoarthritis and rheumatoid arthritis. In a recent study in which matrix metalloproteinases associated with tuberculosis were examined in relation to gender, Sathyamoorthy et al. found that while plasma MMP-8 concentrations inversely correlated with body mass index, they were significantly higher in males than in females [67]. The authors pointed out that this significant difference in gender expression of MMP-8 was not associated with or due to disease severity. Indeed, they concluded that plasma analysis of MMP-1 and MMP-8 was a better discriminator for tuberculosis in men than in women. In a more recent study, it was shown that plasma MMP-3 was significantly higher in men compared to women in a number of clinical conditions that included both infectious and noninfectious diseases including those rheumatic in nature [68].

## 9. Conclusion

Expression of MMPs is ubiquitous characteristic of development. Adherently high expression of MMPs is associated with a host of diseases—infectious and noninfectious. They are particularly significant in the progression and severity of osteoarthritis regardless of etiology. This attests to their utility as major biomarkers for osteoarthritis and mediators of joint destruction. It is worthy to note that MMP-13 is most notably associated with osteoarthritis and once its expression is elevated in the joint significant damage is imminent and the progression to joint destruction is rapid. Present medical knowledge does not provide a way to reverse damage to cartilage caused by osteoarthritis regardless of the insulting modality. It is highly likely that pharmacologic intervention of osteoarthritis will target upstream of MMP-13 expression.

The present short-term treatment for osteoarthritis is pain management, typically in the form of opiates and nonsteroidal anti-inflammatory drugs. While effective at improving the quality of life for osteoarthritis sufferers for a time, the long-term health consequences are severe, and in spite of such interventions joint replacement is almost invariably necessary eventually. There is much opportunity for research leading to an understanding of the processes upstream of the expression of MMP-13.
